# IGF-I induces upregulation of DDR1 collagen receptor in breast cancer cells by suppressing MIR-199a-5p through the PI3K/AKT pathway

**DOI:** 10.18632/oncotarget.6524

**Published:** 2015-12-09

**Authors:** Roberta Matà, Chiara Palladino, Maria Luisa Nicolosi, Anna Rita Lo Presti, Roberta Malaguarnera, Marco Ragusa, Daniela Sciortino, Andrea Morrione, Marcello Maggiolini, Veronica Vella, Antonino Belfiore

**Affiliations:** ^1^ Endocrinology, Department of Health Sciences, University Magna Graecia of Catanzaro, Catanzaro, Italy; ^2^ Department of Biomedical and Biotechnological Sciences Biology, Genetics and BioInformatics Unit, University of Catania, Catania, Italy; ^3^ Department of Urology and Biology of Prostate Cancer Program, Kimmel Cancer Center, Thomas Jefferson University, Philadelphia, PA, USA; ^4^ Department of Pharmacy, Health and Nutritional Sciences, University of Calabria, Rende, Italy; ^5^ Motor Sciences, School of Human and Social Sciences, “Kore” University of Enna, Enna, Italy; ^6^ Department of Clinical and Molecular Bio-Medicine, Endocrinology Unit, University of Catania, Garibaldi-Nesima Medical Center, Catania, Italy

**Keywords:** IGF-IR, insulin-like growth factor-I receptor, DDR1, breast cancer

## Abstract

Discoidin Domain Receptor 1 (DDR1) is a collagen receptor tyrosine-kinase that contributes to epithelial-to-mesenchymal transition and enhances cancer progression. Our previous data indicate that, in breast cancer cells, DDR1 interacts with IGF-1R and positively modulates IGF-1R expression and biological responses, suggesting that the DDR1-IGF-IR cross-talk may play an important role in cancer.

In this study, we set out to evaluate whether IGF-I stimulation may affect DDR1 expression. Indeed, in breast cancer cells (MCF-7 and MDA-MB-231) IGF-I induced significant increase of DDR1 protein expression, in a time and dose dependent manner. However, we did not observe parallel changes in DDR1 mRNA. DDR1 upregulation required the activation of the PI3K/AKT pathway while the ERK1/2, the p70/mTOR and the PKC pathways were not involved. Moreover, we observed that DDR1 protein upregulation was induced by translational mechanisms involving miR-199a-5p suppression through PI3K/AKT activation. This effect was confirmed by both IGF-II produced by cancer-associated fibroblasts from human breast cancer and by stable transfection of breast cancer cells with a human IGF-II expression construct. Transfection with a constitutively active form of AKT was sufficient to decrease miR-199a-5p and upregulate DDR1. Accordingly, IGF-I-induced DDR1 upregulation was inhibited by transfection with pre-miR-199a-5p, which also impaired AKT activation and cell migration and proliferation in response to IGF-I.

These results demonstrate that, in breast cancer cells, a novel pathway involving AKT/miR-199a-5p/DDR1 plays a role in modulating IGFs biological responses. Therefore, this signaling pathway may represent an important target for breast cancers with over-activation of the IGF-IR axis.

## INTRODUCTION

The IGF system has a crucial role in the regulation of mammalian development and growth [1, 2, 3]. Notably, the two main receptors of the system, the type I IGF-I receptor (IGF-IR) and the homolog insulin receptor (IR), which occurs in two isoforms (IR-A and IR-B), are often overexpresssed in cancer cells and may affect not only the early phases of carcinogenesis but also cancer progression and cancer resistance to therapies [4–9]. Their cognate ligands, IGF-I and IGF-II, are frequently abundant in the tumor microenvironment as in fact they can be secreted by the tumor stroma and/or by malignant cells [10, 11]. While IGF-I binds only to the IGF-IR with high affinity and insulin binds only to IR isoforms, IGF-II has high affinity for both the IGF-IR and IR-A, which is the IR isoform preferentially expressed in cancer cells [12, 13].

In the last decade, the IGF-IR has been considered the most appropriate target to hinder IGF system deregulation in cancer, as the IGF-IR is mainly involved in survival and proliferation control, and, unlike the IR, only marginally involved in glucose regulation [8]. However, the recent results of phase 2 and phase 3 trials with IGF-IR blocking agents have been largely disappointing [14–16].

Recently, we found that discoidin domain receptors (DDR1 and DDR2) are candidate molecular partners of IR-A [17] and IGF-IR [18]. DDR1 and DDR2 are encoded by different genes, and belong to a membrane receptor family with tyrosine-kinase activity that bind to and are activated by various forms of collagen [19–21]. DDR1 is widely expressed in normal epithelium, while DDR2 is expressed in stromal and smooth muscle cells. DDRs have 13–15 tyrosine residues in their cytoplasmic domain, which serve as binding sites of Src-homology-2 (SH2) and phosphotyrosine binding (PTB) domain-containing molecules [22].

Interestingly, in breast cancer cells and transfected fibroblasts, DDR1 appears to be an important regulator of IGF-IR expression, trafficking and signaling [18], and increases the expression of IGF-IR protein, thus potentiating its biological activities. These properties are not dependent upon DDR1 action as collagen receptor [18]. These data are in agreement with the notion that IGF-IR and DDR1 play similar roles not only during development but also in cancer [23, 24]. In fact, both IGF-IR and DDR1 are overexpressed in various malignancies, and may sustain epithelial-to-mesenchymal transition, invasion, metastases, as well as cancer resistance to therapies [4–9, 24, 25]. The observed crosstalk between the IGF-IR and DDR1 may have, therefore, important implications in development and cancer progression.

In our present work we asked whether ligand-stimulated IGF-IR might, in turn, affect DDR1 expression. Indeed, in breast cancer cells, exposure to IGFs induced upregulation of DDR1 protein through a PI3K/AKT/miR-199a-5p signaling cascade. Thus, this pathway appears to induce a feed-forward mechanism able to sustain IGF-IR-dependent biological responses.

## RESULTS

### IGF-I causes DDR1 protein upregulation in breast cancer cells

The study was conducted on MCF-7, and MDA-MB-231 human breast cancer cells. Both these cell lines have ductal characteristics and metastatic potential, and respond to IGF-I. MCF-7 cells are estrogen receptor positive and express high DDR1 and IGF-IR levels [18]. In contrast, MDA-MB-231 have characteristics of triple negative cells and express lower levels of both DDR1 and IGF-IR [18].

**Figure 1:**
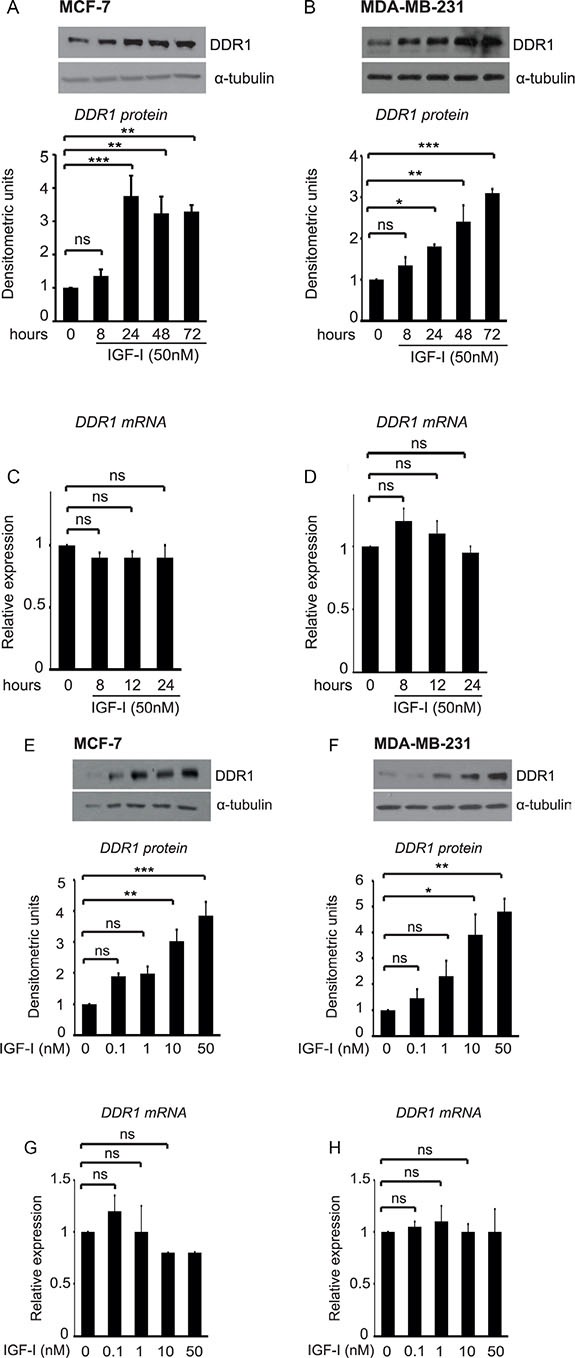
IGF-I induces DDR1 upregulation in breast cancer cells (**A–D**) Time course of DDR1 upregulation after IGF-I. MCF-7 and MDA-MB-231 cells were starved for 24 h, and then stimulated with IGF-I 50 nM for the indicated time points. For DDR1 protein expression (A–B) cells were lysed and DDR1 protein expression was measured by western blotting using a polyclonal antibody against the C-terminus of DDR1. Immunoblot for α-tubulin was used as control for protein loading. For DDR1 mRNA expression (C–D), DDR1 mRNA levels were evaluated by qRT-PCR analysis and values were normalized using S9 as housekeeping control gene. (**E–H**) Dose-response of DDR1 expression after IGF-I. MCF-7 and MDA-MB-231 cells were starved for 24 h, and then stimulated with IGF-I at the indicated doses for 24 h. Western blot for DDR1 protein expression and qRT-PCR analysis for DDR1 mRNA expression were performed as described in (A–D) and in Methods. Each blot is representative of three independent experiments. Values shown in graphs are mean ± SEM of three separate experiments. *, *P* < 0.05; **, *P* < 0.001; ***, *P* < 0.0001.

Both in MCF-7 and MDA-MB-231 cells, IGF-I stimulation significantly upregulated DDR1 protein, in a time and dose dependent manner. DDR1 protein increased as early as 8 h after incubation with IGF-I and reached the maximum of 3–4 folds after 24–72 hours incubation (Figure 1A–1B). In contrast, steady-state DDR1 mRNA levels showed non-significant changes after IGF-I stimulation (Figure 1C–1D), suggesting that translational and/or post-translational mechanisms might have a major role in DDR1 protein upregulation.

**Figure 2:**
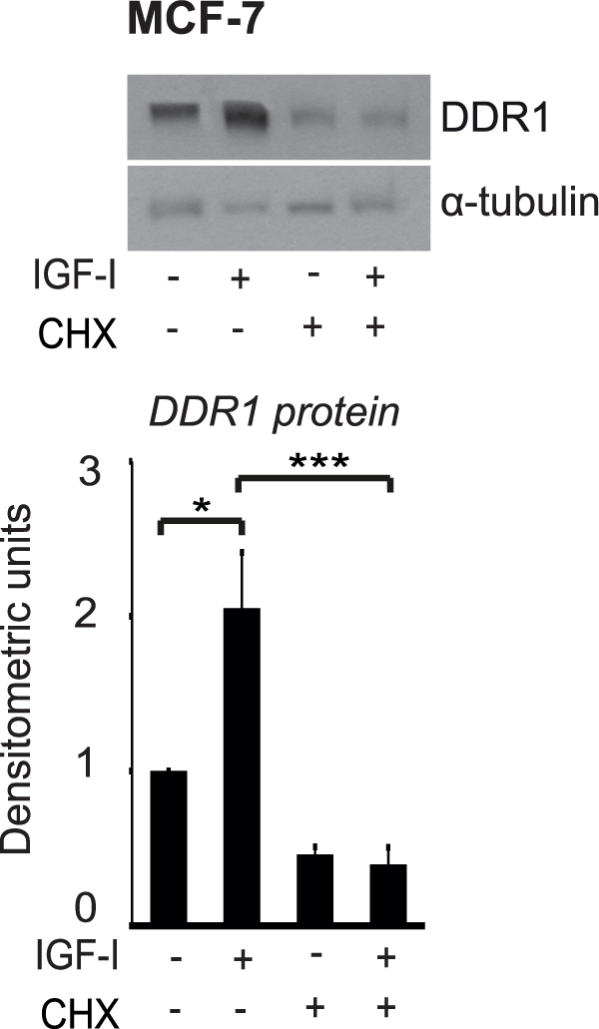
Protein synthesis is involved in DDR1 protein upregulation induced by IGF-I *Cycloheximide studies*. MCF-7 cells were starved for 24 h, pretreated with 10 μg of cycloheximide (CHX) for 1 h and then stimulated with 50 nM of IGF-I for 24 h. Samples were analyzed by western blotting for DDR1 expression using a polyclonal antibody against the C-terminus of DDR1. Immunoblot for α-tubulin antibody was used as control for protein loading. A representative blot of three independent experiments is shown. *P < 0.05; ***P < 0.0001.

Dose-response experiments, carried out at 24 h, showed that, in both cell lines, 1 nM IGF-I already induces noticeable upregulation of DDR1 protein, which reached a maximum with 10–50 nM of IGF-I (Figure 1E–1F). In contrast, DDR1 mRNA levels showed non-significant changes even at higher doses of IGF-I (Figure 1G–1H). Treatment of MCF-7 cells with 10 μM cycloheximide, an inhibitor of translational elongation, completely blocked IGF-I-dependent DDR1 upregulation, suggesting that this process is dependent on new protein synthesis and confirming that translation mechanisms have a role in enhancing DDR1 protein levels (Figure 2).

We then asked whether DDR1 could be also upregulated by other ligands of the IGF system. In MCF-7 cells, which are sensitive to insulin stimulation, DDR1 was upregulated also by IGF-II and insulin (Supplementary Figure 1A), confirming the previously observed crosstalk between DDR1 and the insulin receptor [17]. In MDA-MB-231 cells, we also observed a DDR1 response to IGF-II and insulin stimulation (Supplementary Figure 1B), although these responses were more transient. This is consistent with previous observations that MDA-MB-231 cells express an inhibitor of the IR tyrosine kinase [26, 27], which may regulate the temporal kinetics of IR activation.

### IGF-I-induced DDR1 upregulation is dependent upon the activation of the PI3K/AKT signaling cascade but not the ERK1/2, the mTOR or the PKC cascades

**Figure 3:**
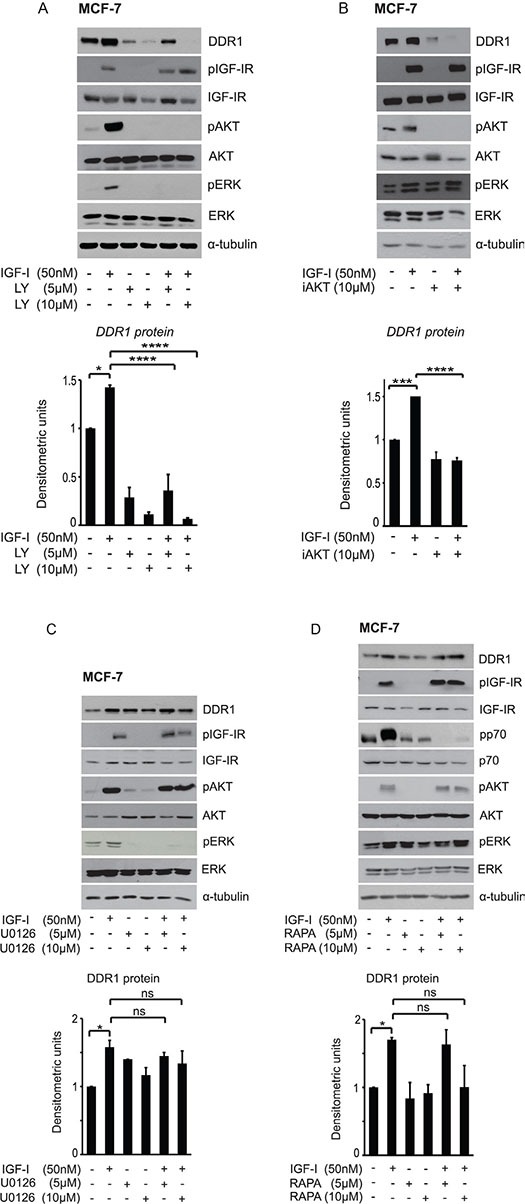
IGF-I dependent DDR1 protein upregulation is downstream the PI3K/AKT pathway and does not require activation of the ERK1/2 and the mTOR pathways MCF-7 cells grown with serum stripped 2.5% of FCS for 24 h, were pretreated with various kinase inhibitors at the indicated doses for 1 h. Cells were then stimulated with 50 nM IGF-I for 24 h, lysed and analyzed by western blotting to evaluate DDR1 protein expression. (**A**) Cells treated with the PI3K inhibitor LY294002 (LY), and (**B**) the AKT inhibitor1/2 (iAKT). (**C**) Cells treated with the MEK1 inhibitor U0126, and (**D**) with the TORC1 inhibitor Rapamycin (Rapa). Immunoblot for α-tubulin was used as control for protein loading. Each blot shown is representative of three independent experiments. Values are mean ± SEM of three separate experiments. *P < 0.05; ***P < 0.0001; ****P < 0.00001.

In MCF-7 cells DDR1 protein upregulation induced by IGF-I was completely blocked by the phosphatidylinositol-3-kinase (PI3K) inhibitor LY-294002 (5–10 μM) (Figure 3A), and by the AKT inhibitor AKT inhibitor1/2 (10 μM) (Figure 3B), while it was not affected by treatment with either the MEK1 inhibitor U0126 (5–10 μM) (Figure 3C) or the mTOR inhibitor rapamycin (5–10 μM) (Figure 3D). Inhibition of the protein Kinase C (PKC) with the PKC ζ myristoylated pseudosubstrate (P9103-71, 5 μM) or with the broad-range PKC inhibitor BIM (1-10 μM) was again ineffective in modulating DDR1 levels (data not shown). These data indicate that the regulation of DDR1 protein levels by IGF-I requires downstream activation of the PI3K/AKT pathway, and that the ERK1/2, the mTOR/p70S6K and the PKC cascades do not play a role in this process.

### IGF-I induces DDR1 protein upregulation by inhibiting miR-199a-5p expression

**Figure 4:**
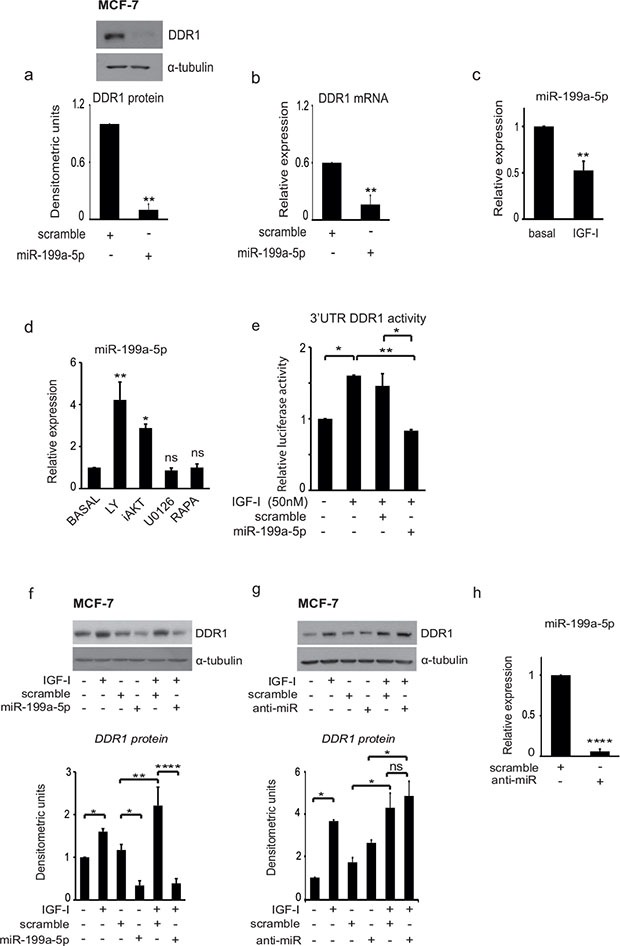
IGF-I induces DDR1 upregulation and increases DDR1 3′UTR activity by inhibiting miR-199a-5p expression (**A–B**) MiR-199a-5p downregulates DDR1. MCF7 cells cultured in complete medium were transfected with 100 nM of pre-miR-199a-5p or scrambled oligonucleotides, as negative control, for 48 h. (A) DDR1 protein expression was determined by western immunoblotting using a polyclonal antibody against the C-terminus of DDR1. Immunoblot for α-tubulin was used as control for protein loading. A representative blot of three independent experiments is shown. Graph represents densitometric values (mean ± SEM) of three separate experiments. **P < 0.005. (B) DDR1 mRNA expression was measured by qRT-PCR analysis and values were normalized using RNU6B as housekeeping control gene. Values are mean ± SEM of three separate experiments. *P < 0.05; **P < 0.005. (**C**) MiR-199a-5p levels are decreased by IGF-I. MCF-7 cells were starved for 24 h and then stimulated for further 24 h with IGF-I (50 nM). MiR-199a-5p levels were measured by qRT-PCR analysis and normalized against RNU6B as housekeeping control gene. Values are mean ± SEM of three separate experiments. **P < 0.005. (**D**) Inhibition of the PI3K/AKT pathway upregulates miR-199a-5p. MCF-7 cells cultured in medium supplemented with 50 nM IGF-I were incubated for 24 h with LY294002, AKT inhibitor1/2 (iAKT), U0126, Rapamycin (Rapa) at a dose of 10 μM. MiR-199a-5p levels were measured by qRT-PCR analysis and normalized using RNU6B as housekeeping control gene. Values are mean ± SEM of three separate experiments. *P < 0.05; **P < 0.001. (**E**) MiR-199a-5p interacts with the 3′UTR of DDR1. MCF-7 cells were co-transfected with a firefly luciferase construct containing the 3′UTR of DDR1 and with pre-miR-199a-5p or control scrambled oligonucleotides (scr). Twenty-four hours after transfection, cells were stimulated with IGF-I (50 nM) for 24 h. The firefly luciferase activity was normalized to protein absorbance. The data are shown as relative luciferase activity of miR-199a-5p-transfected cells as compared to the control (scramble). Values are mean ± SEM of three separate experiments performed in triplicate. *P < 0.05; **P < 0.001. (**F–G**) Effects of pre-miR-199a-5p and anti-miR-199a-5p on DDR1 upregulation by IGF-I. MCF-7 cells were transfected with 100 nM of pre-miR-199a-5p, anti-miR-199a-5p (anti-miR), or scrambled oligonucleotides as negative control for 24 h, starved for 24 h, and then stimulated with 50 nM of IGF-I for further 24 h. Samples were analyzed by western blotting using a polyclonal antibody against the C-terminus of DDR1. Immunoblot for α-tubulin was used as control for protein loading. A representative blot of three independent experiments is shown. Graph represents mean ± SEM of densitometric values of three separate experiments. *P < 0.05; **P < 0.001; ****P < 0.00001. (**H**) Effect of transfection with a specific anti-miR on miR199a5p expression. MCF-7 cells were transfected with 100nM of anti-miR or negative control (scramble) for 48 h. qRT-PCR analysis showed strong inhibition of miR-199a-5p expression. Normalization was done using RNU6B as housekeeping control gene. Values are mean ± SEM of three separate experiments. ****P < 0.00001.

We then evaluated the hypothesis that a regulatory miR could be involved in controlling DDR1 levels. Previous work has reported that in leukemia [28] and in hepatoma cells [29] decreased miR-199a-5p was associated with DDR1 upregulation. We first established that transfection of MCF-7 cells with pre-miR-199a-5p causes a significant reduction of DDR1 protein (Figure 4A) and mRNA (Figure 4B). We then evaluated, whether IGF-I might affect miR-199a-5p levels in MCF-7 cells. Indeed, IGF-I exposure for 24 h caused a significant reduction of miR-199a-5p expression levels, as assessed by qRT-PCR analysis (Figure 4C).

In order to identify the mechanism by which IGF-I regulates miR-199a-5p expression, MCF-7 cells cultured in the presence of 50 nM IGF-1 were incubated for 24 h with various kinase inhibitors, and miR-199a-5p expression levels measured. MiR-199a-5p expression was increased in the presence of inhibitors of both the PI3K and AKT. In contrast, inhibitors of MEK1 and mTOR (TORC1) were ineffective (Figure 4D).

In order to assess whether miR-199a-5p targeted DDR1 at its 3′UTR, and whether this action was affected by IGF-I, we co-transfected MCF-7 cells with pre-miR-199a-5p and a wild type DDR1 3′UTR–luciferase construct. IGF-I increased the DDR1 3′UTR luciferase activity while miR-199a-5p blocked both basal and IGF-I stimulated luciferase activity (Figure 4E). Accordingly, transfection with pre-miR-199a-5p completely blocked IGF-I-induced upregulation of DDR1 protein levels (Figure 4F), which were instead increased by transfection with anti-miR-199a-5p (Figure 4G). As expected, transfection with anti-miR-199a-5p almost completely inhibited miR-199a-5p expression (Figure 4F).

### IGF-I decreases miR-199a-5p and upregulates DDR1 through the PI3K/AKT pathway

**Figure 5:**
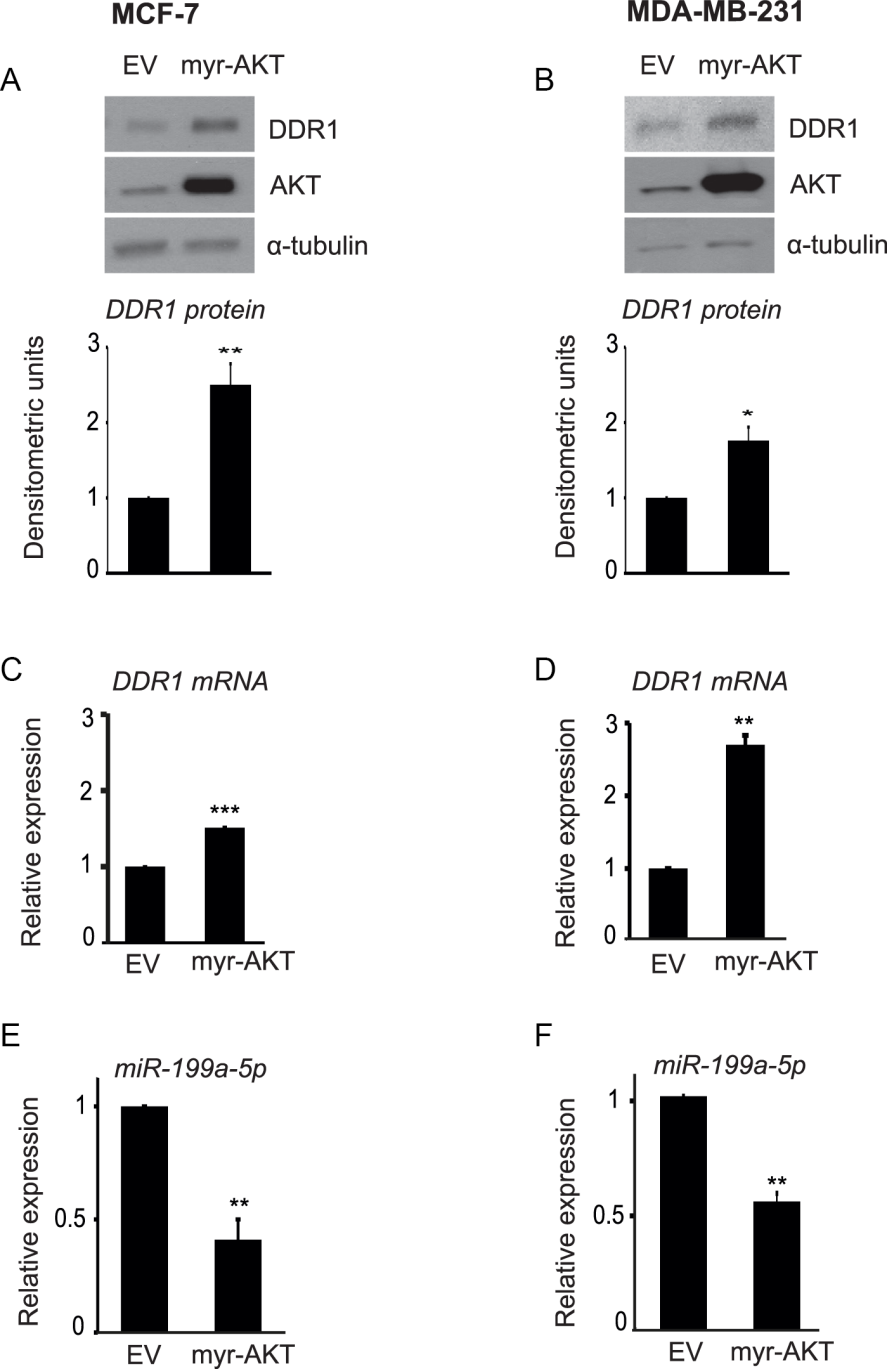
AKT activity is directly involved in DDR1 upregulation and inhibition of miR-199a-5p (**A–D**) Transfection with myr-AKT upregulates DDR1. MCF-7 and MDA-MB-231 cells were transfected with 4.0 μg of pcDNA.3.1-HA-myr-AKT dominant active construct (myr-AKT) or the empty vector pcDNA3.1 (EV) for 48 h and then analyzed by immunoblot using a polyclonal antibody against the C-terminus of DDR1. The filters were blotted for total AKT protein to evaluate transfection efficiency. Immunoblot for α-tubulin was used as control for protein loading. DDR1 mRNA levels (C–D) were evaluated by qRT-PCR analysis and values were normalized using S9 as housekeeping control gene. The experiment shown is representative of three independent experiments. Values are mean ± SEM of three separate experiments. *P < 0.05; **P < 0.001; ***P < 0.0001. (**E–F**) Transfection with myr-AKT downregulates miR-199a-5p. In cells transfected as above, miR-199a-5p expression levels were also measured by qRT-PCR analysis and normalized against RNU6B as housekeeping control gene. MiR-199a-5p was significantly decreased in myr-AKT transfected cells. Values are mean ± SEM of three separate experiments. *P < 0.05; **P < 0.001.

To further corroborate these findings indicating a crucial role of AKT, we transfected both MCF-7 and MDA-MB-231 cells with an active form of AKT (myr-AKT) or a control empty vector. Cells transfected with myr-AKT showed a significant upregulation of DDR1 protein (Figure 5A–5B) and DDR1 mRNA (Figure 5C–5D), which were associated with a marked decrease in miR-199a-5p expression levels (Figure 5E–5F). Collectively, these data confirm that active AKT plays a key role in regulating the inhibition of miR-199a-5p and consequent DDR1 upregulation.

### Autocrine and paracrine IGFs are able to upregulate DDR1 protein in breast cancer cells

**Figure 6:**
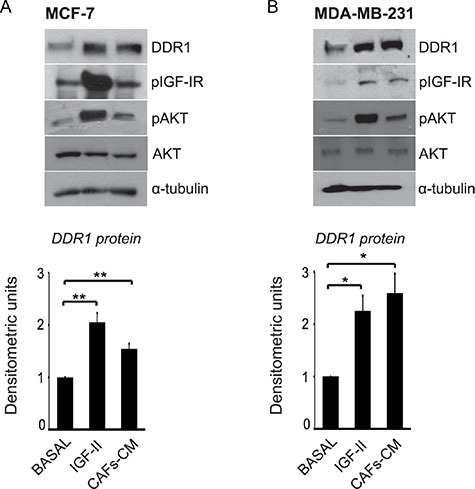
IGF-II from human CAFs-CM is able to upregulate DDR1 protein in breast cancer cells (**A–B**) CAFs-CM upregulates DDR1. MCF-7 and MDA-MB-231 cells were incubated with conditioned medium (1:3, v/v) from human breast cancer CAFs (CAFs-CM). Both in MCF-7 and MDA-MB-231 cells incubation with CAFs-CM for 24 h induced activation of IGF-IR and AKT and a significant upregulation of DDR1. Each blot is representative of three independent experiments. Values shown in graphs are mean ± SEM of three independent experiments. *P < 0.05; **P < 0.001.

Both IGF-I and IGF-II are often produced in an autocrine/paracrine manner in the tumor microenvironment. In particular, breast cancer cells produce autocrine IGF-II, while cancer-associated fibroblasts (CAFs) may produce IGF-I and/or IGF-II [30]. In order to mimic these *in vivo* conditions, we incubated MCF-7 and MDA-MB-231 cells with conditioned medium obtained from CAFs derived from human breast cancers (CAFs-CM). CAFs expression of IGF-I and IGF-II was documented by qRT-PCR (Supplementary Figure 2). Moreover, the concentration of IGF-II protein in CAFs-CM resulted 1.9 ± 0.02 ng/ml, as measured by a specific ELISA. In both cell lines incubation with CAFs-CM for 24 h induced a significant DDR1 upregulation, which was associated with the activation of IGF-IR and AKT (Figure 6A–6B).

**Figure 7:**
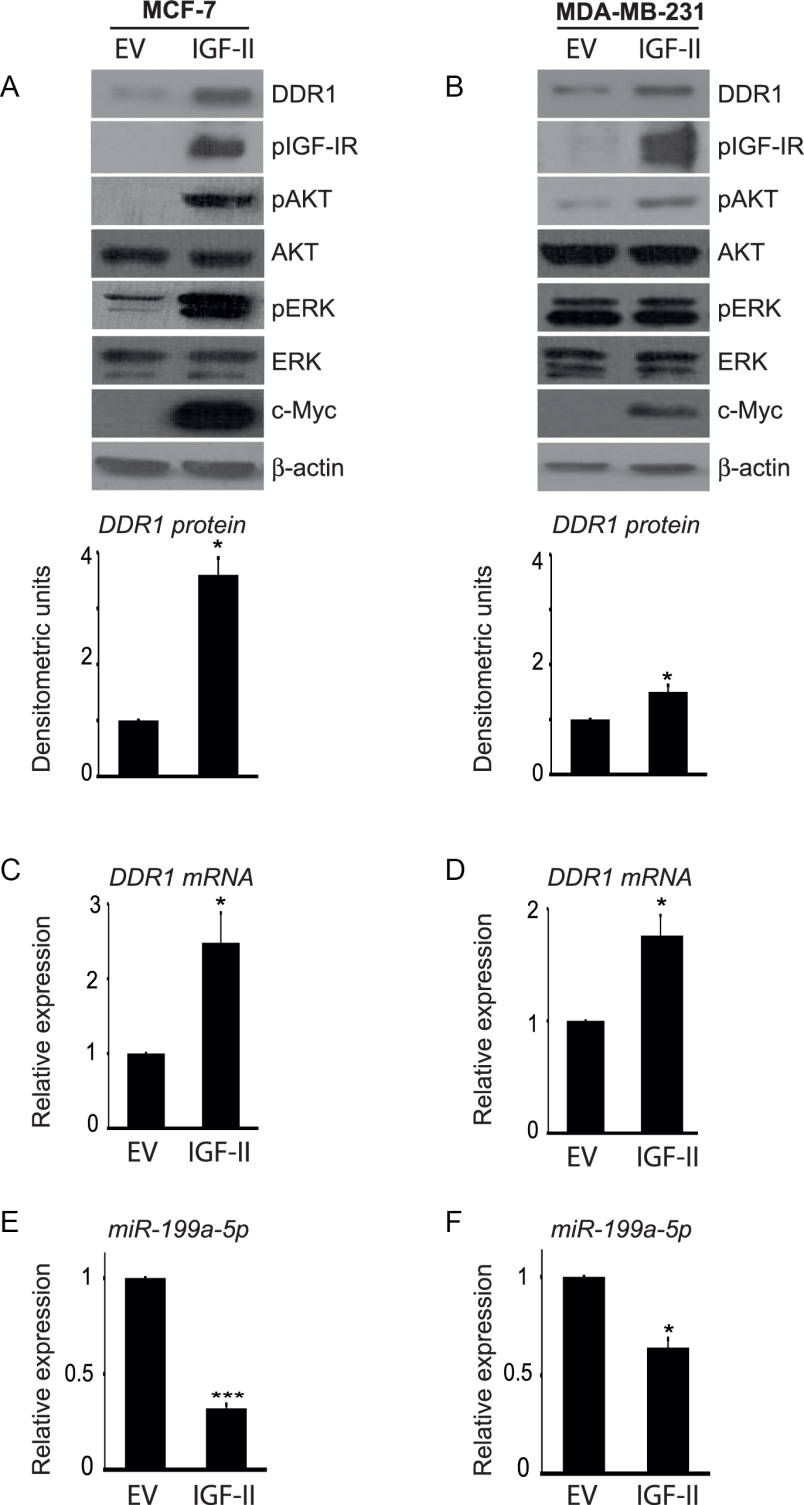
Autocrine IGF-II markedly upregulates DDR1 in breast cancer cells (**A–B**) Constitutively active AKT and ERK1/2 and DDR1 upregulation in IGF-II transfected cells. (A) MCF-7 and (B) MDA-MB-231 cells were transfected with a c-Myc tagged IGF-II expression construct (IGF-II) or a control empty vector (EV). Cells were solubilized and samples analyzed by western blotting with anti-DDR1, phospho-IGF-IR, phospho-S473-AKT and phospho-ERK1/2 antibodies. The same blots were probed with anti-AKT, anti-ERK1/2, anti-β-actin, and anti-c-Myc antibodies to check for protein loading and cell transfection efficiency. Both cell lines showed constitutively active IGF-IR, AKT and ERK1/2 and significant increase in DDR1 protein and DDR1 mRNA (**C–D**) in IGF-II transfected cells. Each blot is representative of three independent experiments. Values shown in graphs are mean ± SEM of three independent experiments. *P < 0.05. (**E–F**) Inhibition of miR-199a-5p in IGF-II transfected cells. In cell transfected as above, miR-199a-5p expression levels significantly decreased. Values shown in graphs are mean ± SEM of three independent experiments. *P < 0.05; ***P < 0.0001.

Moreover, we stably transfected MCF-7 and MDA-MB-231 cells with an IGF-II expression construct in order to induce elevated levels of autocrine IGF-II production. Transfected cells showed high production of IGF-II, as assessed by qRT-PCR (not shown), constitutive IGF-IR autophosphorylation and a high degree of AKT and ERK1/2 activation (Figure 7A–7B). Importantly, both in MCF-7 and MDA-MB-231 cells autocrine IGF-II production determined a significant increase in DDR1 protein (Figure 7A–7B) and mRNA levels (Figure 7C–7D), which were associated with decreased miR-199a-5p expression levels (Figure 7E–7F).

Taken together, these data strongly suggest that paracrine/autocrine IGFs production in cancer may result in increased DDR1 expression through the activation of a PI3K/AKT/miR-199a-5p pathway.

**Figure 8:**
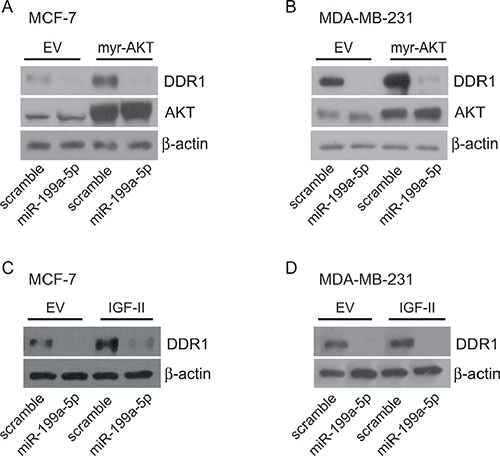
miR199a-5p inhibits DDR1 up-regulation induced by myr-AKT or autocrine IGF-II MCF-7 and MDA-MB-231 cells, stably transfected with either pcDNA3.1HA-myr-AKT, control pcDNA3.1HA (**A**–**B**), or with c-Myc tagged IGF-II expression construct or the relative empty vector (**C–D**), were starved for 24 h and then transfected with 100 nM miR199a-5p or scramble oligonucleotides for 48 h. Cells were then evaluated by western blotting for DDR1 expression with a polyclonal antibody against the C-terminus of DDR1. In myr-AKT transfected cells AKT expression was also evaluated. β-actin was used as control for protein loading.

In order to demonstrate that DDR1 downregulation by miR-199a-5p is relevant also in cells expressing myr-AKT or autocrine IGF-II, MCF-7 and MDA-MB-231 cells stably expressing either myr-AKT or autocrine IGF-II were transfected with miR-199a-5p. As shown in Figure 8, transfection with miR-199a-5p determined a strong inhibition of DDR1 expression levels in those cells.

### MiR-199a-5p inhibits proliferation and migration of breast cancer cells

**Figure 9:**
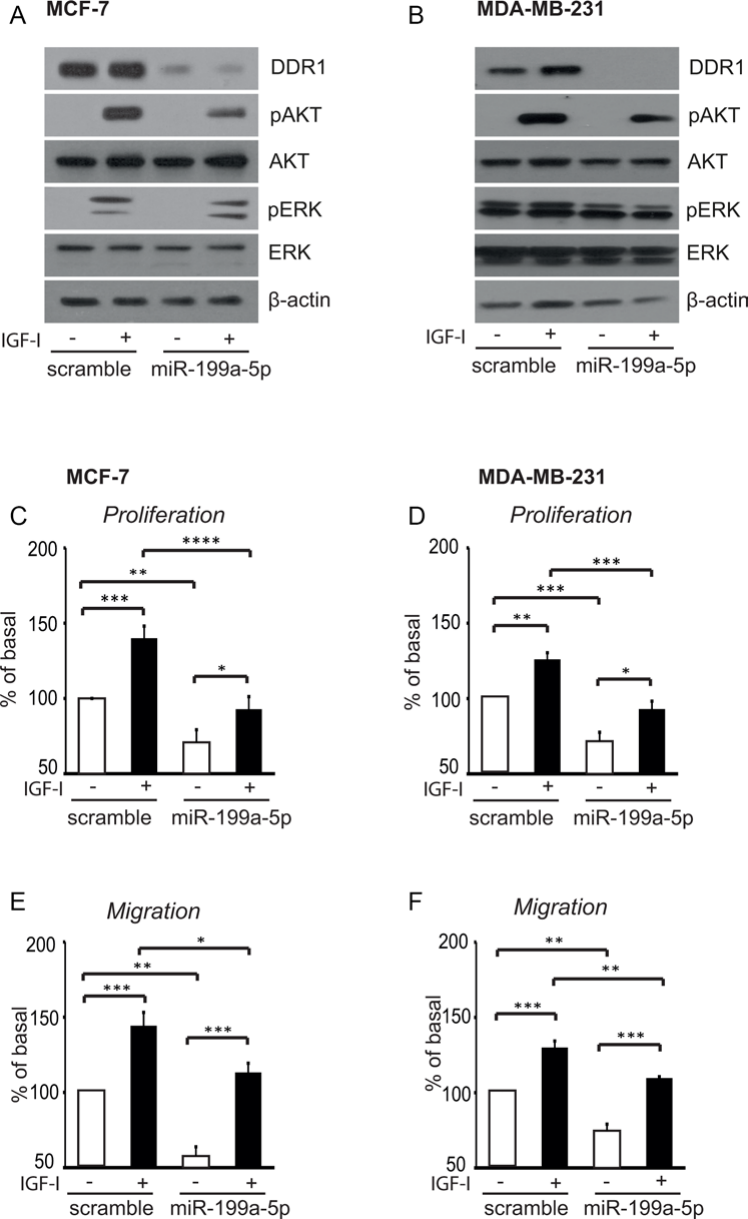
In breast cancer cells miR-199a-5p inhibits IGF-I signaling, and cell proliferation and migration (**A–B**) IGF-I signaling in cells transfected with pre-miR199a-5p. MCF-7 and MDA-MB-231 cells were transfected with 100 nM of pre-miR-199a-5p, or negative control for 24 h, starved for 24 h and then stimulated with 50 nM of IGF-I for 5 min. Cells were solubilized and samples analyzed by western blotting with phospho-S473-AKT and phospho-ERK1/2 antibodies. The same blots were probed with anti-AKT, anti-ERK1/2 and anti-β-actin antibodies to check for protein loading. (**C–F**) Biological responses to IGF-I in cells transfected with pre-miR199a-5p. (C, E) MCF-7 and (D, F) MDA-MB-231 cells transfected and cultured as above were stimulated with 50 nM of IGF-I for further 24 h. Cell viability was measured by the methyl thiazolyl tetrazolium (MTT) assay, and cell migration through fibronectin-coated filters was evaluated in Boyden chambers. Values are mean ± SEM of three separate experiments. *P <0.05; **P < 0.001; ***P < 0.0001;****P < 0.00001.

We previously showed that, in breast cancer cells, DDR1 silencing by small RNA interference impairs IGF-I induced downstream signaling and biologic responses [18]. We now evaluated whether DDR1 dowregulation induced by miR-199a-5p was also able to affect IGF-I signaling and biological effects. Indeed, transfection of MCF-7 and MDA-MB-231 cells with pre-miR-199a-5p significantly downregulated DDR1, and reduced the activation of both the AKT and ERK1/2 cascades in response to IGF-I (Figure 9A, 9B). Both in MCF-7 (Figure 9C, 9E) and in MDA-MB-231 cells (Figure 9D, 9F) pre-miR-199a-5p also significantly inhibited proliferation and cell migration through fibronectin-coated filters in response to IGF-I.

**Figure 10:**
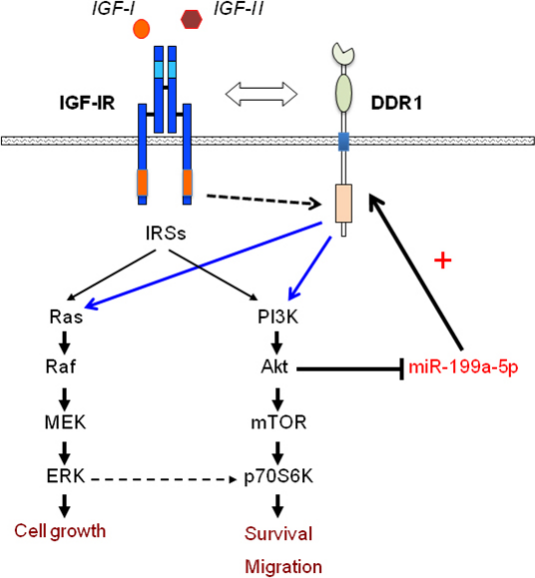
Schematic representation of the positive feedback involving IGF-IR expression and function through the AKT/miR-199a-5p/DDR1 pathway AKT activation through paracrine/autocrine IGFs induces miR-199a-5p inhibition, which in turn causes DDR1 upregulation. As previously reported [18], the crosstalk with DDR1 regulates IGF-IR trafficking and expression, and enhances IGFs downstream signaling and biological responses.

Taken together, these data indicate that miR-199a-5p suppression via autocrine/paracrine IGFs and AKT activation causes upregulation of DDR1, which enhances IGFs signaling and biological responses, thereby delineating a new mechanism by which IGFs enhance their own biological responses (Figure 10).

### Computational analysis of microarray gene expression in human cells and tissues

We then asked whether DDR1 expression was positively correlated with the IGF-IR and/or with the homologous receptors insulin receptor (INSR or IR), and/or with IGF-IR ligands *in vivo*. To this end, as illustrated in Methods, we sought for these correlations across several conditions and numerous cell and tissues types using the Affymetrix GeneChip Human Genome U133 plus 2.0 arrays (consisting of 1794 datasets) and the betaMEM method. We validated the most statistically relevant MEM results in The Cancer Genome Atlas (TCGA) breast cancer dataset. By MEM expression analysis, we found DDR1 to be strongly and positively correlated with IGF-IR, IGFBP2, IGFBP5 and AKT expression. A moderate positive correlation was also found between DDR1 and INSR (Table 1). The positive correlation of DDR1 with IGF-IR and IGFBP2, but not with AKT, INSR or IGFBP5, was further confirmed in the TCGA breast cancer dataset (Table 1). Moreover, DDR1 expression negatively correlated with DDR2, and weakly correlated with PTEN (Table 1). No significant correlation was found between DDR1 and IGF-I or IGF-II expression (not shown). Conflicting data (or below our stringent *p* value's cutoff) were found instead when we correlated DDR1 expression levels with IGFBP1, IGFBP2 (not shown).

**Table 1:** Correlations between three different probes for DDR1 and components of the IGF system

	betaMEM scores			TCGA breast
Gene Probes +	DDR1_208779_X_AT	DDR1_207169_X_AT	DDR1_210749_X_AT	Pearson coefficient (*P*-value)
IGF1R_225330_at	1.37E-19	3.83E-20	1.12E-20	0.29 *(< 0.00001)*
IGF1R_203628_at	6.66E-13	7.03E-13	2.24E-13	
IGF1R_203627_at	7.04E-10	4.36E-09	5.52E-10	
IGF1R_243358_at	2.28E-07	2.48E-06	2.60E-06	
INSR_226450_at	5.79E-08	1.62E-08	2.68E-08	0.09 (0.035)
INSR_213792_s_at	9.07E-07	2.50E-08	4.42E-07	
AKT1_207163_s_at	4.52E-10	1.71E-10	9.42E-11	0.069 (0.15)
IGFBP2_202718_at	5.6E-20	1.03E-19	2.44E-20	0.18 (< 0.00001)
IGFBP5_211959_at	5.60E-20	6.79E-14	1.69E-16	0.025 (0.47)
IGFBP5_211958_at	3.23E-15	6.76E-10	5.97E-10	
IGFBP5_203425_s_at	3.64E-10	1.48E-06	6.37E-06	
IGFBP5_203424_s_at	9.67E-07	2.17E-06	9.54E-07	
IGFBP5_1555997_s_at	1.28E-06	1.29E-05	2.56E-05	
Gene Probes −				
DDR2_227561_at	4.86E-15	9.14E-13	4.37E-17	−0.26 *(< 0.00001)*
DDR2_205168_at	8.17E-14	7.87E-14	1.17E-17	
DDR2_225442_at	3.65E-13	1.21E-10	4.37E-15	
DDR2_235631_at	6.13E-11	6.18E-08	2.34E-11	
PTEN_228006_at	1.25E-07	5.74E-06	8.46E-07	−0.03 *(0.48)*

Statistically significant correlations between three different probes for DDR1 (i.e., 208779_X_AT, 207169_X_AT, 210749_X_AT) and probes for components of the IGF system, according to betaMEM scores ≤ 1 × 10^−5^. Pearson coefficients and *P*-values of equivalent genes from TCGA breast analysis are reported in the last column. Gene probes (+), probes positively correlated; Gene probes (−), probes negatively correlated.

Next, we evaluated whether miR-199a-5p was negatively correlated with DDR1 or with IGF-IR in a panel of human cancer histotypes. Indeed, StarBase Pan-Cancer analysis showed a significant inverse correlation between miR-199a-5p and DDR1 expression in 8/14 cancer histotypes. In addition, miR-199a-5p also inversely correlated with IGF-IR in 2/14 cancer types (Table 2).

**Table 2:** Anticorrelations of expression between miR-199a-5p and DDR1 and IGF-IR across 14 cancer types from TCGA

	DDR1		IGF-IR	
Cancer Type	Pearson coefficient	*P*-value	Pearson coefficient	*P*-value
Urothelial bladder cancer	−0.03	*0.64*	0.06	*0.3*
Breast cancer	−**0.17**	***0.000001***	0.11	*0.002*
Colon and Rectal adenocarcinoma	−0.05	*0.3*	0.17	*0.002*
Glioblastoma multiforme	−**0.37**	***0.000001***	0.07	*0.33*
Head and neck squamous cell carcinoma	−**0.11**	***0.01***	0.009	*0.84*
Chromophobe renal cell carcinoma	−**0.66**	***0.000000000001***	−**0.43**	***0.00001***
Clear cell kidney carcinoma	−0.05	*0.36*	0.07	*0.21*
Acute Myeloid Leukemia	0.13	*0.08*	0.05	*0.5*
Lung adenocarcinoma	0.12	*0.01*	0.03	*0.46*
Lung squamous cell carcinoma	0.04	*0.42*	0.06	*0.21*
Ovarian serous cystadenocarcinoma	−**0.19**	***0.001***	0.007	*0.89*
Cutaneous melanoma	−**0.22**	***0.00003***	−**0.18**	***0.0004***
Papillary thyroid carcinoma	−**0.11**	***0.008***	0.05	*0.16*
Uterine corpus endometrial carcinoma	−0.3	*0.0001*	0.17	*0.02*

Analysis was performed according to starBase Pan-Cancer Platform. Bold typed data are statistically significant.

Collectively, these data strongly support our *in vitro* data and provide strong evidence that IGF-IR activity may induce DDR1 upregulation through decrease of miR-199a-5p *in vivo* human breast cancer.

## DISCUSSION

We have previously reported that, in breast cancer cells and in transfected fibroblasts, the collagen receptor DDR1 associates with the IGF-IR in an IGF-I dependent manner [18]. This DDR1–IGF-IR interaction leads to increased IGF-IR expression, signaling, and enhanced mitogenic and migratory response to IGF-I stimulation. In murine fibroblasts transfected with the human IGF-IR, the concomitant overexpression of DDR1 markedly enhanced IGF-IR protein expression and IGF-I-dependent colony formation. Both in breast cancer cells and transfected fibroblasts, IGF-I stimulation induced rapid tyrosine-phosphorylation of DDR1, which was accompanied by increased association of the DDR1 – IGF-IR complex, rapid internalization and sorting to early endosomes [18].

Herein we found that, in breast cancer cells, exposure to IGF-I induced significant upregulation of DDR1 protein, which was not accompanied by concomitant similar changes in DDR1 mRNA expression. DDR1 protein upregulation was time- and dose-dependent following IGF-I exposure, and required IGF-IR tyrosine kinase activity, as it was abolished by incubation with the specific IGF-IR tyrosine kinase inhibitor NVP-AWE-541 (not shown). DDR1 upregulation was dependent upon the activation of the PI3K/AKT pathway, while the ERK1/2 and the mTOR/p70S6K or the PKC pathways were not implicated. Accordingly, DDR1 upregulation was abolished by inhibitors of PI3K and AKT, and was reproduced by transfection with myr-AKT, a constitutively activated form of AKT. In order to further dissect the mechanisms underlying DDR1 upregulation, we found that IGF-I, via activation of the PI3K/AKT pathway, induced miR-199a-5p downregulation, which, in turn, increased DDR1 protein expression.

Indeed, transfection of breast cancer cells with miR-199a-5p markedly downregulated DDR1 protein while miR-199a-5p antagomir upregulated it. DDR1 mRNA showed less marked changes than DDR1 protein. Accordingly, treatment of breast cancer cells with miR-199a-5p blunted IGF-I signaling and inhibited breast cancer cell proliferation and migration in response to IGF-I.

MiRNAs are endogenous 18–24 nucleotide single-stranded RNA molecules that regulate posttranscriptional gene expression by inhibiting protein translation or inducing mRNA degradation [31]. miRNAs interact with the 3′-untranslated region (3′-UTR) of target mRNAs, and the resulting effects depends on the degree of complementarity of each miRNA to the target mRNA. Perfect complementarity results in mRNA degradation and gene silencing, while partial complementarity results in modest targeted mRNA degradation. The final effect may also depend on the set of proteins bound to the 3′-UTR of the target mRNA [31]. Previously, *DDR1* has been reported to be a target gene of miR-199a-5p, which shows only partial complementarity to the 3′UTR of DDR1 mRNA [29]. Here we confirm that, in breast cancer cells, miR-199a-5p reduces the activity of a luciferase construct containing the 3′-UTR of the DDR1 mRNA. However, this effect of miR-199a-5p is significantly reduced by IGF-I stimulation

Interestingly, miR-199a-5p downregulation caused by cell exposure to IGF-I was associated with significant changes of DDR1 protein but limited changes of DDR1 mRNA. In contrast, cells transfection with myr-AKT or IGF-II expression constructs, caused more marked upregulation of DDR1 mRNA. Our present results are in agreement with previous work demonstrating an inverse relationship between DDR1 and miR-199a-5p. For instance, transfection of miR-199a-5p in hepatoma cells significantly down-regulated DDR1 protein but did not induce changes in DDR1 mRNA expression [29]. However, our observation that IGF-I upregulates DDR1 through miR-199a-5p downregulation via the PI3K/AKT pathway is novel and not previously reported. Nevertheless, our data are in accordance with the results of Rane et al. indicating that activated AKT is sufficient to downregulate miR-199a-5p in cardiac myocytes [32]. Hypoxia has been also been reported to reduce miR-199a-5p, possibly by inducing AKT activation through the integrin-linked kinase, ILK [33, 34]. Thus, AKT activation seems a nodal upstream regulator of the miR-199a-5p - DDR1 pathway common to both IGF-I and hypoxia. Data obtained in cardiac myocytes suggest that hypoxia-depend miR-199a-5p downregulation does not require transcriptional activity [35]. Therefore, it is likely that miR-199a-5p regulation by the IGF-IR/AKT pathway is also posttranscriptional and involves miRNAs' processing and/or stability.

Common events of IGF system dysregulation in cancer involve receptor overexpression as well as autocrine/paracrine expression of IGF-I and IGF-II [30, 36]. We showed that incubation with the conditioned medium from CAFs from human breast cancer was able to activate IGF-IR, AKT, and upregulate DDR1 in breast cancer cells. Moreover, stable transfection of breast cancer cells with the *IGF-II* gene resulted in constitutively activated AKT, reduced miR-199a-5p levels, and markedly upregulated DDR1 expression. Notably, these cells, which had very high AKT activity showed significant upregulation of both DDR1 protein and mRNA.

Taken together, these results indicate that, in breast cancer, paracrine/autocrine IGFs are positive modulators of DDR1 expression, and help explaining the frequent DDR1 overexpression in a variety of malignancies with dysregulated IGF axis.

In view of our previous results indicating that DDR1 and IGF-IR form a complex that enhances IGF-I effects in cancer cells [18], our present findings suggest that the IGF-IR/AKT/miR-199a-5p/DDR1 pathway is an important feed-forward mechanism for enhancing IGF-IR effects (Figure 10). We can therefore hypothesize that DDR1 upregulation, by enhancing IGF-IR stability, may counteract IGF-IR downregulation by autocrine expression of IGFs. Indeed, the marked overexpression of functional receptors for insulin and IGF-I in most malignant cells with concomitant autocrine IGFs secretion suggest that ligand-dependent receptor down-regulation and degradation may be impaired.

Importantly, by bioinformatics approach, we were able to highlight a positive correlation between IGF-IR and DDR1 in expression data from several databases including the TCGA (The Cancer Genome Atlas) that comprises 522 primary tumors, 3 metastatic tumors, and 22 tumor-adjacent normal samples. We found a positive correlation between DDR1 and other components of the IGF system, such as AKT, IGF-BP2, IGF-BP5 and IR. Moreover, miR-199a-5p inversely correlated with DDR1 expression in several tumors including cancers from breast, lung, ovary, thyroid, endometrium and acute myeloid leukemia. These data confirm the biological relevance of this IGF-IR/AKT/miR-199a-5p/DDR1 axis.

The correlation between DDR1 and IR and the finding that insulin may also upregulate DDR1, raise the possibility that insulin resistance and compensatory hyperinsulinemia may enhance DDR1 in neoplastic tissues. Further studies are needed to confirm this possibility and to fully understand the clinical relevance of this effect.

Notably, IGF-I and IGF-II were also able to upregulate DDR1 in triple negative-like MDA-MB-231 cells, which are known to be responsive to IGF-I [18, 37]. However, MDA-MB-231 were less responsive to insulin, in agreement to the fact that they overexpress PC1/ENPP1, an inhibitor of the IR tyrosine kinase [26, 27], which may reduced the intensity of IR activation.

Overactivation of the IGF pathways appears to play a fundamental role in cell stemness both in normal and cancer tissues through multiple mechanisms [38]. Notably, both IGF-IR and DDR1 have been implicated in the induction and maintenance of epithelial-mesenchymal transition (EMT), one hallmark of stem cells and a critical process allowing tissue remodeling during embryogenic and tumor invasion and metastasis [39]. MiR-199a-5p downregulation itself may also play a role in EMT by causing E-cadherin loss [40]. Therefore, our present data may suggest that the activation of the IGF-IR/AKT/miR-199a-5p/DDR1 pathway may play a relevant role in cancer EMT, cell stemness, invasion and metastasis. Moreover, overactivation of IGF system in cancer is also increasingly recognized as associated to resistance to various anticancer therapies [16, 41−43]. For these reasons, in the last decade, much effort has been devoted to target the IGF-IR in cancer either with blocking antibodies or with small molecules with tyrosine kinase inhibiting activity [14]. However, results have been largely disappointing, because of the emergence of various and not well-characterized mechanisms of resistance [41, 16, 44, 15, 45]. We hypothesize that the activation of the AKT/miR-199a-5p/DDR1 pathway may represent one of these mechanisms of resistance and that it could be a suitable target in malignancies associated with dysregulated IGF system. Importantly, our studies raise the possibility that DDR1 inhibitors [46, 47] may synergize with IGF-IR blocking strategies. Further studies are needed therefore to confirm these hypotheses.

## MATERIALS AND METHODS

### Materials

IGF-I and IGF-II were from Preprotech (Rocky Hill, NJ), lipofectamine 2000 and lipofectamine RNAiMax from Life Technologies Inc. Laboratories (Paisley, UK), scramble (miR-NC), synthetic pre-miR-199a-5p (miR-199a-5p), and mirVana miR-199a-5p inhibitor were purchased from Ambion (Applied Biosystems, CA, USA), fibronectin from Sigma-Aldrich (Saint Louis Missouri, USA).

Constructs encoding either pcDNA.3.1-HA-myr-AKT dominant active construct (AKT) or the empty vector pcDNA3.1 (vector) were kindly provided by prof. P. Tassone (University “Magna Graecia” of Catanzaro).

Construct encoding 3′UTR clone of DDR1 in pMirTarget Vector, the related empty vector (pCMV6) and the human IGF-II cDNAs were from OriGene Technologies (Rockville, USA). The following kinase inhibitors were used: the IGF-IR inhibitor NVP-AEW541 (Cayman Chemical, Ann Arbor, USA); the PI3 kinase inhibitor LY 294002 (Calbiochem, Merck Millipore, Nottingham, UK); the MEK1 inhibitor U0126 (Sigma-Aldrich, Saint Louis Missouri, USA); the TORC1 inhibitor rapamycin (Sigma-Aldrich); the AKT 1,2 inhibitor (Sigma-Aldrich, Saint Louis Missouri, USA). Actinomycin D and cycloheximide were purchased from Sigma-Aldrich, MTT from Amersham Bioscences.

### Cell cultures and conditioned medium analysis

The human cancer cell lines MCF-7, MDA-MB-231 were purchased from the American Cell Type Culture Collection, and cultured according to the manufacturer's instructions. Cells were grown in MEM (Sigma-Aldrich) supplemented with 10% fetal bovine serum (Gibco, Life Technologies, Carlsbad, CA, USA). Cancer-associated fibroblasts (CAFs) from human breast cancer were established as previously described [48] and grown in Medium-199 (Life Technologies) supplemented with 10% fetal bovine serum. Conditioned medium from CAFs (CAFs-CM) was prepared by incubating CAFs in serum-free medium for 48 h. IGF-II concentration in CAFs-CM was measured by a commercially available ELISA kit (E30 Mediagnost, Reutlingen, Germany; minimum detectable concentration: 0.02 ng/ml).

### Western blot Analysis

For dose-response analysis, subconfluent cells were incubated in serum-deprived medium for 24 h, stimulated with increasing ligand concentrations for 24 h at 37°C and solubilized in radioimmune precipitation (RIPA) buffer; for time-course experiments, subconfluent cells were treated with IGF-I for the indicated time points.

To evaluate IGF-I - dependent activation of DDR1, cells were serum-starved for 24 h, and then stimulated with IGF-I (50 nM) for 24 h.

Cell lysates were subjected to western blot analysis, as previously described [49]. The following antibodies were used: anti-DDR1 (C-20, sc-532, Santa Cruz Biotechnology), anti-phospho(p)-IR/p-IGF-IR (Y1150/Y1151, 3024, Cell Signaling), anti-IGF-IR (9750, Cell Signaling), anti-p-AKT (Ser473, 9271, Cell Signaling), anti-AKT(9272, Cell Signaling), anti-p-ERK1/2 (T202/Y204, 4370, Cell Signaling), anti-ERK1/2 (9102, Cell Signaling), anti-p-p70 (Thr421/Ser424, 9204, Cell Signaling); anti-p70 (C-18, 230 Santa Cruz Biotechnology); anti-α-tubulin (15246, Abcam); anti-actin (Sigma).

### Real-time PCR

Total cellular RNA was extracted using TRIzol Reagent (Invitrogen, Life Technologies, Carlsbad, CA, USA) and miRNAs were isolated with microRNA Purification Kit (Norgen Biotek, Thorold Ontario, Canada) according to the manufacturer's protocol. qRT–PCR was used to confirm the expression levels of mRNAs and miRNAs. Total RNA (1 μg) was reversely transcribed using the ThermoScript RT (Invitrogen) and oligo (dT) primers. Total microRNA (10 ng) was reversely transcribed using TaqMan MicroRNA Reverse Transcription Kit (Applied Biosystems), and synthesized cDNA was analyzed in a PCR reaction using primers for the gene of interest, as previously described [50]. qRT-PCR reactions were performed in a StepOne Plus Real-Time PCR System (Applied Biosystems). The ΔΔCt method of relative quantification and SYBR Green chemistry were used to measure DDR1 mRNA (DDR1 all FW: 5′-GCGTCTGTCTGCGGGTAGAG-3′, RV: 5′-ACGGCCTCAGATAAATACATTGTCT-3′), IGF-I mRNA (FW: 5′-TCGCATCTCTTCTATCTGGCCCTGT-3′, RV: 5′-GCAGTACATCTCCAGCCTCCTCAGA-3′) and IGF-II mRNA (FW: 5′-GACCGCGGCTTCTACTTCAG-3′, RV: 5′-AAGAACTTGCCCACGGGGTAT-3′). S9 and 18S were used as endogenous controls for normalization (S9, FW: 5′-CTGGGTTTGTCGCAAAACTT-3′; RV: 5′-GTGGGTCCTTCTCATCAAGC-3′) (18S, FW: 5′-AGGAATTCCCAGTAAGTGCG-3′ RV: 5′-GCCTCACTAAACCATCCAA-3′). The single-tube TaqMan miRNA (Assay ID 000498, Applied Biosystems, Life Technologies) was used to detect and quantify mature miR-199a-5p according to the manufacturer's instructions. MiR-199a-5p expression was normalized against RNU6B expression (Assay ID 001093, Applied Biosystems).

### Gene transfection

For microRNA experiments, cells were transfected with a mixture containing OptiMEM, RNAimax and either 100 nM miR-199a-5p or scramble oligonucleotides. For gene overexpression experiments, cells were transfected with a mixture containing the DNA of interest, OptiMEM and Lipofectamine 2000. Twenty-four hours after transfection, cells were serum-starved for 24 h and then treated with IGF-I (50 nM) for 24 h.

*IGF-II* transfected cellular clones were generated by transfecting MCF-7 and MDA-MB231 cells with pCMV6-Entry vector and the pCMV6-containing the human *IGF-II* gene. After transfection, cells were selected in G-418 containing medium and subcloned.

### Luciferase assay

MCF-7 cells were transiently transfected, as described above, with 1 μg of the firefly luciferase reporter plasmid containing the 3′UTR of DDR1 cloned in pMIR-target vector. Twenty-four hours after transfection, cells were incubated with fresh medium and transfected with 100 nM of the synthetic miR-199a-5p or scramble oligonucleotides using Lipofectamine 2000. Cells were stimulated with IGF-I 50 nM for additional 24 h. Firefly luciferase activity was detected using the dual-luciferase assay Kit (Promega Corporation, Madison, WI, USA) 48 h after transfection. The luciferase activity was normalized to protein absorbance.

### Cell viability

MCF-7 and MDA-MB-231 cell lines were plated in 48 wells plates in standard culture medium. After 24 h, cells were transfected using 100 nM of miR-199a-5p or scramble oligonucleotides, serum-starved for 24 h, and then stimulated with IGF-I 50 nM for additional 24 h. Cell viability was then measured by the methyl thiazolyl tatrazolium (MTT) test, according to the manufacturer's instructions. Briefly, the cells were incubated with medium containing 0.5-mg/mL MTT; after 4 h, the cells were dissolved in 100 μL of a solution containing dimethyl sulfoxide plus 2.5% complete medium, and formazan absorbance was read at 405 nm.

### Migration assay

The ability of cells to invade through fibronectin was measured with Boyden's chamber technique as previously described [18, 51]. Cells, serum starved for 24 h, were placed on polycarbonate filters (8 μm pore size, Corning Costar) coated on the upper side with 25 mg/mL fibronectin. Filters were placed over bottom chambers containing serum-free medium with or without ligand (50 nM). After incubation for 3–6 h, depending on the cell type, cells on the upper surface of filters were removed with a cotton swab, and the filters were stained for 20 min with crystal violet (0.05% crystal violet in PBS plus 20% ethanol). After three washes with water, crystal violet was solubilized in 10% acetic acid for 30 min at room temperature, and its concentration was evaluated by absorbance at 595 nm.

### Computational analysis of microarray gene expression

We analyzed the correlation of expression of DDR1 with DDR2 and with critical receptors and ligands of the IGF system (i.e., IGF-IR, INSR, AKT, IGF-I, IGF-II, PTEN, IGFBP1, IGFBP2, IGFBP3, IGFBP4, IGFBP5) across many conditions and many cell and tissues types by using the online tool Multi Experiment Matrix (MEM) (http://biit.cs.ut.ee/mem/index.cgi). MEM is a repository of large collections of microarray datasets that performs statistical significance estimation of distances (positive and negative expression correlations) by Pearson correlation distance; a *p*-value on the ranks was determined using the betaMEM method. We selected Affymetrix GeneChip Human Genome U133 plus 2.0 arrays (consisting of 1794 datasets) for our analysis. We considered betaMEM scores ≤ 1 × 10^−5^ as statistically significant. Moreover, we validated the most statistically relevant MEM results on The Cancer Genome Atlas (TCGA) breast cancer dataset. These latter data were obtained from the TCGA breast cancer online portal (https://tcga-data.nci.nih.gov/docs/publications/brca_2012/). Specifically, we computed Pearson coefficient between genes from the microarray gene expression data file “BRCA.exp.547.med.txt” that consists of 522 primary tumors, 3 metastatic tumors, and 22 tumor-adjacent normal samples. Data were median centered by genes. For this analysis, Pearson coefficients with a stringent *P*-values ≤ 0.00001 were considered statistically significant. Data of expression correlation between miR-199a-5p and genes from the IGF system were retrieved from starBase Pan-Cancer Platform by analysis of expression profiles of 14 cancer types from TCGA Data Portal [PMID: 24297251].

### Densitometric and Statistical analysis

Densitometry results were obtained by using NIH ImageJ. Differences between means were evaluated by one-way ANOVA followed by post-hoc analysis of significance (Bonferroni test) for the comparison between more than two groups, whereas the Student's *t* test for unpaired samples was used for comparisons between two groups. The level of significance was set at *P* < 0.05. Statistical analysis was performed with GraphPad Prism6 (GraphPad Software, San Diego, CA). Data were expressed as mean ± SEM.

## SUPPLEMENTARY MATERIALS FIGURES



## References

[1] Brogiolo W, Stocker H, Ikeya T, Rintelen F, Fernandez R, Hafen E (2001). An evolutionarily conserved function of the Drosophila insulin receptor and insulin-like peptides in growth control. Curr Biol.

[2] Stewart CE, Rotwein P (1996). Growth, differentiation, and survival: multiple physiological functions for insulin-like growth factors. Physiol Rev.

[3] Bartke A (2005). Minireview: role of the growth hormone/insulin-like growth factor system in mammalian aging. Endocrinology.

[4] Belfiore A, Frittitta L, Costantino A, Frasca F, Pandini G, Sciacca L, Goldfine ID, Vigneri R (1996). Insulin receptors in breast cancer. Ann N Y Acad Sci.

[5] Carboni JM, Lee AV, Hadsell DL, Rowley BR, Lee FY, Bol DK (2005). Tumor development by transgenic expression of a constitutively active insulin-like growth factor I receptor. Cancer Res.

[6] Baserga R, Peruzzi F, Reiss K (2003). The IGF-1 receptor in cancer biology. Int J Cancer.

[7] Yakar S, Leroith D, Brodt P (2005). The role of the growth hormone/insulin-like growth factor axis in tumor growth and progression: Lessons from animal models. Cytokine Growth Factor Rev.

[8] Belfiore A, Frasca F, Pandini G, Sciacca L, Vigneri R (2009). Insulin receptor isoforms and insulin receptor/insulin-like growth factor receptor hybrids in physiology and disease. Endocr Rev.

[9] Novosyadlyy R, Lann DE, Vijayakumar A, Rowzee A, Lazzarino DA, Fierz Y (2010). Insulin-mediated acceleration of breast cancer development and progression in a nonobese model of type 2 diabetes. Cancer Res.

[10] Sciacca L, Costantino A, Pandini G, Mineo R, Frasca F, Scalia P, Sbraccia P, Goldfine ID, Vigneri R, Belfiore A (1999). Insulin receptor activation by IGF-II in breast cancers: evidence for a new autocrine/paracrine mechanism. Oncogene.

[11] Paik S (1992). Expression of IGF-I and IGF-II mRNA in breast tissue. Breast Cancer Res Treat.

[12] Frasca F, Pandini G, Scalia P, Sciacca L, Mineo R, Costantino A, Goldfine ID, Belfiore A, Vigneri R (1999). Insulin receptor isoform A, a newly recognized, high-affinity insulin-like growth factor II receptor in fetal and cancer cells. Mol Cell Biol.

[13] Belfiore A, Malaguarnera R (2011). Insulin receptor and cancer. Endocr Relat Cancer.

[14] Baserga R (2013). The decline and fall of the IGF-I receptor. J Cell Physiol.

[15] King H, Aleksic T, Haluska P, Macaulay VM (2014). Can we unlock the potential of IGF-1R inhibition in cancer therapy?. Cancer Treat Rev.

[16] Manara MC, Garofalo C, Ferrari S, Belfiore A, Scotlandi K (2013). Designing novel therapies against sarcomas in the era of personalized medicine and economic crisis. Curr Pharm Des.

[17] Morcavallo A, Gaspari M, Pandini G, Palummo A, Cuda G, Larsen MR, Vigneri R, Belfiore A (2011). Research resource: new and diverse substrates for the insulin receptor isoform a revealed by quantitative proteomics after stimulation with igf-ii or insulin. Mol Endocrinol.

[18] Malaguarnera R, Nicolosi ML, Sacco A, Morcavallo A, Vella V, Voci C, Spatuzza M, Xu SQ, Iozzo RV, Vigneri R, Morrione A, Belfiore A (2015). Novel cross talk between IGF-IR and DDR1 regulates IGF-IR trafficking, signaling and biological responses. Oncotarget.

[19] Vogel W (1999). Discoidin domain receptors: structural relations and functional implications. FASEB J.

[20] Leitinger B (2014). Discoidin domain receptor functions in physiological and pathological conditions. Int Rev Cell Mol Biol.

[21] Borza CM, Pozzi A (2014). Discoidin domain receptors in disease. Matrix Biol.

[22] Lemeer S, Bluwstein A, Wu Z, Leberfinger J, Müller K, Kramer K, Kuster B (2012). Phosphotyrosine mediated protein interactions of the discoidin domain receptor 1. J Proteomics.

[23] Gallagher EJ, LeRoith D (2011). Minireview: IGF, Insulin, and Cancer. Endocrinology.

[24] Valiathan RR, Marco M, Leitinger B, Kleer CG, Fridman R (2012). Discoidin domain receptor tyrosine kinases: new players in cancer progression. Cancer Metastasis Rev.

[25] Shintani Y, Fukumoto Y, Chaika N, Svoboda R, Wheelock MJ, Johnson KR (2008). Collagen I-mediated up-regulation of N-cadherin requires cooperative signals from integrins and discoidin domain receptor 1. J Cell Biol.

[26] Costantino A, Milazzo G, Giorgino F, Russo P, Goldfine ID, Vigneri R, Belfiore A (1993). Insulin-resistant MDA-MB231 human breast cancer cells contain a tyrosine kinase inhibiting activity. Mol Endocrinol.

[27] Belfiore A, Costantino A, Frasca F, Pandini G, Mineo R, Vigneri P, Maddux B, Goldfine ID, Vigneri R (1996). Overexpression of membrane glycoprotein PC-1 in MDA-MB231 breast cancer cells is associated with inhibition of insulin receptor tyrosine kinase activity. Mol Endocrinol.

[28] Favreau AJ, Cross EL, Sathyanarayana P (2012). miR-199a-5p directly targets PODXL and DDR1 and decreased levels of miR-199a-5p correlate with elevated expressions of PODXL and DDR1 in acute myeloid leukemia. Am J Hematol.

[29] Shen Q, Cicinnati VR, Zhang X, Iacob S, Weber F, Sotiropoulos GC, Radtke A, Lu M, Paul A, Gerken G, Beckebaum S (2010). Role of microRNA-199a-5p and discoidin domain receptor 1 in human hepatocellular carcinoma invasion. Mol Cancer.

[30] Sadlonova A, Novak Z, Johnson MR, Bowe DB, Gault SR, Page GP, Thottassery JV, Welch DR, Frost AR (2005). Breast fibroblasts modulate epithelial cell proliferation in three-dimensional *in vitro* co-culture. Breast Cancer Res.

[31] Bartel DP (2004). MicroRNAs: genomics, biogenesis, mechanism, and function. Cell.

[32] Rane S, He M, Sayed D, Yan L, Vatner D, Abdellatif M (2010). An antagonism between the AKT and beta-adrenergic signaling pathways mediated through their reciprocal effects on miR-199a-5p. Cell Signal.

[33] Tan C, Cruet-Hennequart S, Troussard A, Fazli L, Costello P, Sutton K, Wheeler J, Gleave M, Sanghera J, Dedhar S (2004). Regulation of tumor angiogenesis by integrin-linked kinase (ILK). Cancer Cell.

[34] Lee S-P, Youn S-W, Cho H-J, Li L, Kim T-Y, Yook H-S, Park KW, Oh BH, Park YB, Kim HS (2006). Integrin-linked kinase, a hypoxia-responsive molecule, controls postnatal vasculogenesis by recruitment of endothelial progenitor cells to ischemic tissue. Circulation.

[35] Sayed D, Abdellatif M (2010). AKT-ing via microRNA. Cell Cycle.

[36] Sciacca L, Costantino A, Pandini G, Mineo R, Frasca F, Scalia P, Sbraccia P, Goldfine ID, Vigneri R, Belfiore A (1999). Insulin receptor activation by IGF-II in breast cancers: evidence for a new autocrine/paracrine mechanism. Oncogene.

[37] Bartucci M, Morelli C, Mauro L, Ando S, Surmacz E (2001). Differential insulin-like growth factor I receptor signaling and function in estrogen receptor (ER)-positive MCF-7 and ER-negative MDA-MB-231 breast cancer cells. Cancer Res.

[38] Malaguarnera R, Belfiore A (2014). The emerging role of insulin and insulin-like growth factor signaling in cancer stem cells. Front Endocrinol (Lausanne).

[39] Thiery JP, Acloque H, Huang RYJ, Nieto MA (2009). Epithelial-mesenchymal transitions in development and disease. Cell.

[40] Hu Y, Liu J, Jiang B, Chen J, Fu Z, Bai F, Jiang J, Tang Z (2014). MiR-199a-5p loss up-regulated DDR1 aggravated colorectal cancer by activating epithelial-to-mesenchymal transition related signaling. Dig Dis Sci.

[41] Garofalo C, Manara MC, Nicoletti G, Marino MT, Lollini PL, Astolfi A, López-Guerrero JA, Schaefer KL, Belfiore A, Picci P, Scotlandi K (2011). Efficacy of and resistance to anti-IGF-1R therapies in Ewing's sarcoma is dependent on insulin receptor signaling. Oncogene.

[42] Fox EM, Miller TW, Balko JM, Kuba MG, Sanchez V, Smith RA (2011). A kinome-wide screen identifies the insulin/IGF-I receptor pathway as a mechanism of escape from hormone dependence in breast cancer. Cancer Res.

[43] Fox EM, Kuba M, Miller TW, Davies BR, Arteaga CL (2013). Autocrine IGF-I/insulin receptor axis compensates for inhibition of AKT in ER-positive breast cancer cells with resistance to estrogen deprivation. Breast Cancer Res.

[44] Yap TA, Olmos D, Molife LR, de Bono JS (2011). Targeting the insulin-like growth factor signaling pathway: figitumumab and other novel anticancer strategies. Expert Opin Investig Drugs.

[45] Malaguarnera R, Belfiore A (2011). The insulin receptor: a new target for cancer therapy. Front Endocrinol.

[46] Li Y, Lu X, Ren X, Ding K (2015). Small molecule discoidin domain receptor kinase inhibitors and potential medical applications. J Med Chem.

[47] Kothiwale S, Borza CM, Lowe EW, Pozzi A, Meiler J (2015). Discoidin domain receptor 1 (DDR1) kinase as target for structure-based drug discovery. Drug Discov Today.

[48] De Marco P, Romeo E, Vivacqua A, Malaguarnera R, Abonante S, Romeo F, Pezzi V, Belfiore A MM (2014). GPER1 is regulated by insulin in cancer cells and cancer-associated fibroblasts. Endocr. Relat. Cancer.

[49] Sacco A, Morcavallo A, Pandini G, Vigneri R, Belfiore A (2009). Differential signaling activation by insulin and insulin-like growth factors I and II upon binding to insulin receptor isoform A. Endocrinology.

[50] Malaguarnera R, Frasca F, Garozzo A, Giani F, Pandini G, Vella V, Vigneri R, Belfiore A (2011). Insulin receptor isoforms and insulin-like growth factor receptor in human follicular cell precursors from papillary thyroid cancer and normal thyroid. J Clin Endocrinol Metab.

[51] Malaguarnera R, Sacco A, Voci C, Pandini G, Vigneri R, Belfiore A (2012). Proinsulin Binds with High Affinity the Insulin Receptor Isoform A and Predominantly Activates the Mitogenic Pathway. Endocrinology.

