# Supplementary material for: IGF-I induces upregulation of DDR1 collagen receptor in breast cancer cells by suppressing MIR-199a-5p through the PI3K/AKT pathway
Source: Oncotarget. 2015 Dec 9;7(7):7683–700. doi: 10.18632/oncotarget.6524 (PMC4884947; doi:10.18632/oncotarget.6524)
Supplement: Supplementary file 1 [file oncotarget-07-7683-s001.pdf]

IGF-I induces upregulation of DDR1 collagen receptor in breast cancer cells by suppressing MIR-199a-5p through the PI3K/AKT pathway

Supplementary Materials

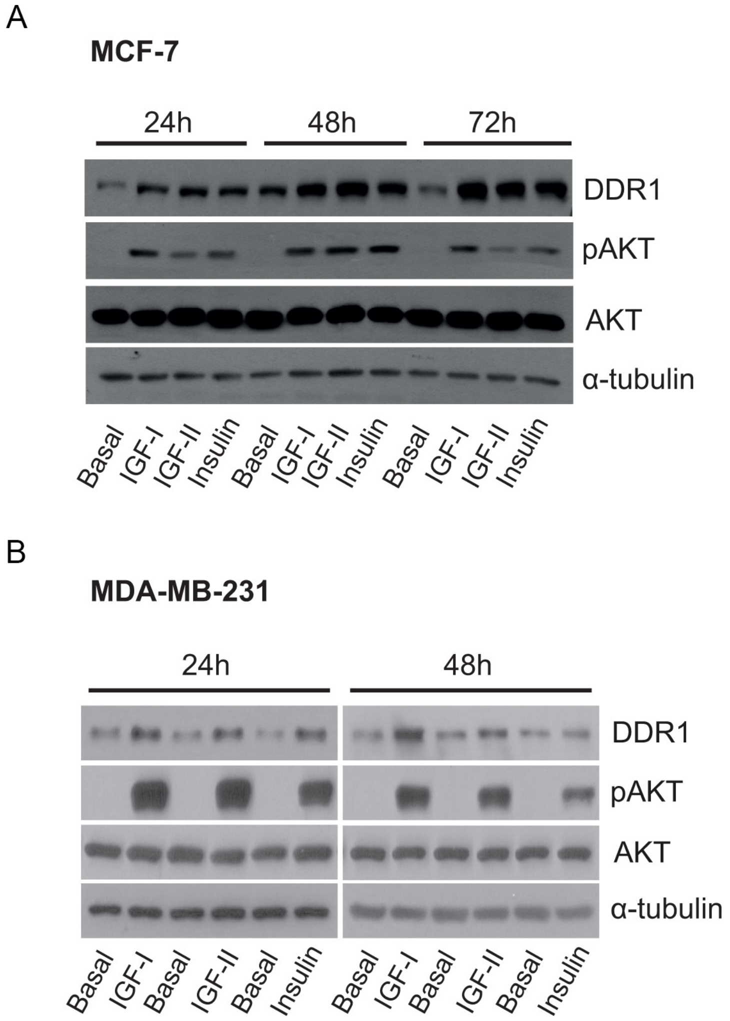

Supplementary Figure 1: Time Course of DDR1 upregulation after IGF-I, IGF-II or Insulin. (**A**) MCF-7 and (**B**) MDA-MB-231 cells were starved for 24 h and then stimulated for the indicate time points with 50 nM of IGF-I, IGF-II or Insulin. DDR1 protein expression was measured by western blotting using a polyclonal antibody against the C-terminus of DDR1. Immunoblot for α-tubulin was used as control for protein loading. Values are mean ± SEM of three separate experiments. $*P < 0.05$; $**P < 0.001$; $***P < 0.0001$.

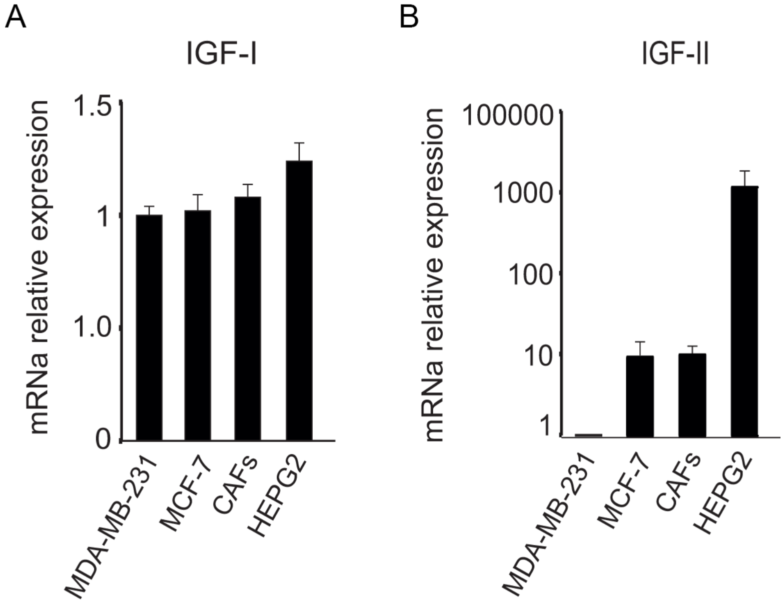

Supplementary Figure 2: IGF-1 and IGF-II mRNA expression in cultured cells. (**A−B**) qRT-PCR analysis of endogenous IGF-I and IGF-II levels in CAFs, as compared with MDA-MB-231, MCF-7, and HEPG2 cells. Normalization was done using 18 s as housekeeping control gene.
